# Evolving Improved Sampling Protocols for Dose–Response Modelling Using Genetic Algorithms with a Profile-Likelihood Metric

**DOI:** 10.1007/s11538-024-01304-1

**Published:** 2024-05-08

**Authors:** Nicholas N. Lam, Rua Murray, Paul D. Docherty

**Affiliations:** 1 Department of Mechanical Engineering, University of Canterbury, Christchurch, New Zealand; 2 School of Mathematics and Statistics, University of Canterbury, Christchurch, New Zealand; 3 Institute of Technical Medicine, Furtwangen University, Villingen-Schwenningen, Baden-Württemberg Germany

**Keywords:** Model-based design of experiments, Practical identifiability, Identifiability, Profile likelihood

## Abstract

Practical limitations of quality and quantity of data can limit the precision of parameter identification in mathematical models. Model-based experimental design approaches have been developed to minimise parameter uncertainty, but the majority of these approaches have relied on first-order approximations of model sensitivity at a local point in parameter space. Practical identifiability approaches such as profile-likelihood have shown potential for quantifying parameter uncertainty beyond linear approximations. This research presents a genetic algorithm approach to optimise sample timing across various parameterisations of a demonstrative PK-PD model with the goal of aiding experimental design. The optimisation relies on a chosen metric of parameter uncertainty that is based on the profile-likelihood method. Additionally, the approach considers cases where multiple parameter scenarios may require simultaneous optimisation. The genetic algorithm approach was able to locate near-optimal sampling protocols for a wide range of sample number (n = 3–20), and it reduced the parameter variance metric by 33–37% on average. The profile-likelihood metric also correlated well with an existing Monte Carlo-based metric (with a worst-case r > 0.89), while reducing computational cost by an order of magnitude. The combination of the new profile-likelihood metric and the genetic algorithm demonstrate the feasibility of considering the nonlinear nature of models in optimal experimental design at a reasonable computational cost. The outputs of such a process could allow for experimenters to either improve parameter certainty given a fixed number of samples, or reduce sample quantity while retaining the same level of parameter certainty.

## Introduction

Parameter identification is the process of determining the optimal values of a set of model parameters to fit the model to observed behaviour (Villaverde and Banga 2014). Parameter identifiability analysis is the process of determining how reliably parameters can be estimated from data. When considering finite data, this is often called practical identifiability analysis, while towards the infinite data limit, this becomes structural identifiability analysis (Simpson et al. 2020)**.** Practical limitations in experimentation such as measurement noise and discrete sampling locations can restrict the information available for the process of parameter identification, potentially leading to practical identifiability issues (Raue et al. 2009; Hines et al. 2014; Wieland et al. 2021; Lam et al. 2022; Muñoz-Tamayo and Tedeschi 2023). Consequently, a wide distribution of parameters may exhibit similar model behaviour and are not distinguishable to measured data. In such cases, the optimised parameter values have low certainty, and thus, the information yielded by the model-based analysis is ambiguous. Such issues have given rise to the model-based design of experiments (MBDoE) approach (also known as Optimal Experimental Design), which aims minimise uncertainty for parameter identification through adjusting experimental design settings (Franceschini and Macchietto 2008).

MBDoE approaches have been developed to address the difficulty of optimising experiments in non-linear models. MBDoE approaches can guide the choice of experimental design elements such as test inputs, experiment duration, and measurement timing. MBDoE has seen extensive research over several decades (Jacquez and Greif 1985; Walter and Pronzato 1990; Franceschini and Macchietto 2008; Galvanin et al. 2013). The approaches have predominantly required a scalar metric to be optimised through the MBDoE process, such as those based on properties of the Fisher information matrix (FIM).

The FIM is a first-order linear approximation of model sensitivity at a nominal parameter set, and it is indicative of the local convexity of the objective surface (Lam et al. 2022). D-optimality criteria, which maximises the determinant of the FIM, has been the most commonly employed metric for MBDoE (Franceschini and Macchietto 2008). Other common metrics are E-optimality and A-optimality, which maximise the smallest eigenvalue and trace of the FIM, respectively. However, as noted by Krausch et al. (2019) and Raue et al. (2009), using the FIM to approximate the accuracy of parameter estimation is not justified in the presence of nonlinearity in a region proximal to the optimised parameter values. Furthermore, optimising designs around a single parameter set can be an issue if multiple characteristic behaviours outside of that parameter domain exist in measured data (Franceschini and Macchietto 2008; Lam et al. 2022). For example, in a disease modelling context, a schedule optimised based on the parameters of a healthy individual may be detrimental to the parameter identification of a sick individual, or vice versa.

Recent developments in MBDoE have focused on tackling the issue of non-linearity in model behaviour. Methods have been developed alongside methods of practical identifiability analysis, since both methods share a goal of improving parameter estimation. In 2019, Krausch et al. (2019) developed a new metric, the Q-criterion, based on quantiles of Monte Carlo simulations performed on the model rather than a measure of the FIM. The Q-criterion was shown to capture non-linearities in a Michaelis–Menten kinetic example when used in conjunction with a MBDoE toolbox. Outside of the traditional MBDoE approach, practical identifiability based methods such as the generalised sensitivity functions developed by Thomaseth and Cobelli (1999), a graphical approach by Docherty et al. (2011), and profile-likelihood (PL) approach popularised by Raue et al. (2010) have been developed to improve experimental design. Of these methods, uptake of the PL approach has been particularly high (Wieland et al. 2021; Lam et al. 2022; Villaverde et al. 2023). Distinct advantages of the PL approach have been its ease of implementation and interpretability, along with computation speeds roughly an order of magnitude lower than comparable Monte Carlo-based methods (Simpson et al. 2020).

As noted by Lin et al. (2015), the use of genetic algorithms (GA) to generate optimal sampling schedules has seen an increase in recent decades. Inspired by evolutionary biology, GAs for MBDoE consider a population of candidate sampling schedules as *organisms*, and these organisms compete in successive generations with a goal of gradual improvement towards a near-optimal solution. The selection process that determines successive generations is reliant on a metric that acts as a *fitness function* to rank the optimality of each organism. In one selection scheme of GA known as the *elitist* variant, the best individuals from a current generation are selected for the next, along with additional individuals that have been created with crossover and/or mutation operations (Lin et al. 2015). However, applications of GA and other stochastic optimisation methods for MBDoE have predominantly used measures based on the FIM such as D-optimality (Broudiscou et al. 1996; Heredia-Langner et al. 2004; Chen et al. 2015). As noted earlier, these measures can miss nonlinear model behaviour.

This paper proposes the use of a profile-likelihood based metric in conjunction with a genetic algorithm to overcome the limitations of both the linear assumptions implicit in FIM-based measures, and the computational burden of Monte Carlo simulations. The proposed methodology is used to determine the optimal sample placement in a simple dose–response experiment with concomitant models of varying complexity. There is literature that describes the relationship between confidence interval width and sample size (Rothman and Greenland 2018). Using confidence interval-based metrics in MBDoE is complicated by the need to consider both sample placement and sample size, and current research on these methods is limited. In the pharmacokinetic-pharmacodynamic (PK-PD) context of this modelling, sampling is often limited by both the physical consequences of drawing multiple blood samples and the cost of analyte measurement in a lab setting (Mori and DiStefano 1979; DiStefano 1981; Docherty et al. 2011; Galvanin et al. 2013). Recent research has highlighted that quantifying uncertainty pharmacological model parameters is challenging due to the range of complexity in prospective models (Sher et al. 2022). The aim of the study is to explore the benefits of optimising sampling schedules in a sparse sampling scenario using a novel method that is rapid and relatively easy to implement.

## Method

### Cases Investigated

A toy model with simple pharmacokinetic-pharmacodynamic behaviour was chosen for testing the GA approach. First-order dynamics for a concentration $C$ are described by

1 $$\begin{array}{*{20}c} {\dot{C}\left( t \right) = - k\frac{C\left( t \right)}{{1 + \beta C\left( t \right)}} + \frac{U_N + U_x \left( t \right)}{V}} \\ \end{array}$$

where $C$ is an arbitrary concentration, and $\dot{C}$ is its time derivative, $k$ is a first-order decay rate, $V$ is the volume of distribution, $U_N$ is the endogenous production rate, and $U_x \left( t \right)$ is the external bolus. The bolus $U_x (t)$ is defined as an instantaneous input at time $t$ = 60 min. For the purposes of testing the experimental design protocol, Case 1 considered ${{\varvec{\theta}}} = \left\{ {k, U_N } \right\}^T$ as unknown parameters to be identified, while $V$ is an a priori value and $\beta = 0$. Case 2 considered ${{\varvec{\theta}}} = \left\{ {k, U_N , V} \right\}^T$ as unknown parameters to be identified with $\beta = 0$. Case 3 considers a model with Michaelis–Menten mechanics (Michaelis and Menten 1913). In this more complex model, ${{\varvec{\theta}}} = \left\{ {k, U_N , V, \beta } \right\}^T$ were non-zero and identified.

**Fig. 1:**
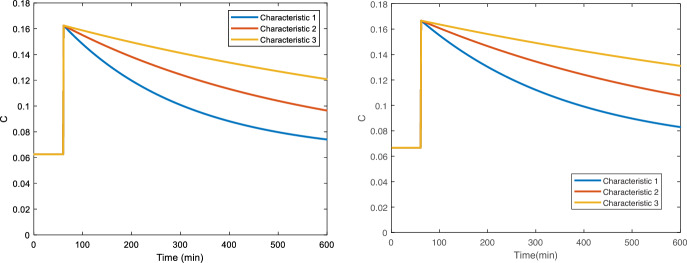
Trajectories of model scenarios used in Cases 1 and 2 (left) and Case 3 (right)

To consider and explore the local nature of optimality in experimental design, three distinct parameter scenarios were considered for each model case (Table 1). Numerical integration of Eq. 1 was performed for 0–600 min, the magnitude of $U_x (t)$ was set as 4.0 for 1 min at $t$ = 60 min. Simulations, shown in Fig. 1, were undertaken using Euler’s method with a step size of 1 min. For all simulated cases and parameter identification, it was assumed that $C_0$ was at equilibrium. The three scenarios were chosen to resemble the progression towards a biological saturated response, where either age or the progression of a disease may inhibit the body’s ability to process an input.

**Table 1:** Values of parameters used for parameter scenarios 1–3 with Eq. (1)

	$k$	$U_N$	$V$	$\beta$(Case 1–2)	$\beta$(Case 3)
Scenario 1	4.0e−3	1.0e−2	40	0	1.0
Scenario 2	2.0e−3	5.0e−3	40	0	1.0
Scenario 3	1.0e−3	2.5e−3	40	0	1.0

### Practical Identifiability Methods

The Q-criterion ($Q_{crit}$) was developed by Krausch et al. (2019) as a measure of average parameter confidence interval width, where

2 $$\begin{array}{*{20}c} {Q_{crit} = \sum \limits_{\theta_i } \left( {Q_{\theta_i , 0.9} - Q_{\theta_i , 0.1} } \right)^2 } \\ \end{array}$$

uses $Q_{\theta_i , 0.9}$ and $Q_{\theta_i , 0.1}$, the 90th and 10th quantile bounds of samples generated by Monte Carlo analysis, respectively. $Q_{crit}$ is summed over each $i^{th}$ parameter within the parameter vector ${{\varvec{\theta}}}$**.** Based on Eq. 2, a general metric for capturing parameter identifiability based on confidence intervals is proposed as

3 $$\begin{array}{*{20}c} {CI_{crit} = \sqrt {{ \sum \limits_{\theta_i } \left( {\frac{\theta_i^+ - \theta_i^- }{{2\hat{\theta }_i }}} \right)^2 }} } \\ \end{array}$$

where $\hat{\theta }_i$ is the best estimate of $\theta_i$ from parameter identification, and $\left[ {\theta_i^- , \theta_i^+ } \right]$ are the confidence intervals of the parameters obtained from a given method. Based on the coefficient of variation, the metric scales the confidence interval width by $\hat{\theta }_i$ to allow for comparison of parameters of differing magnitudes. Minimisation of $CI_{crit}$ results in reduction of average normalised parameter uncertainty. Profile-likelihood was selected as the method to form confidence intervals in this study. Using the profile-likelihood to form confidence intervals has advantages over FIM-based methods: it is invariant under nonlinear transformations and applicable to nonlinear models (Wieland et al. 2021). Additionally, profile-likelihood can identify confidence intervals more efficiently than Monte Carlo simulations, making it computationally advantageous for the methods covered later in Sect. 3.2.

Raue et al. (2009) defined a method for using profile-likelihood to determine confidence intervals for model parameters. Parameters are ‘profiled’ by fixing a single $\theta_i$ along a range of values while fitting the non-fixed $\theta_{j \ne i}$ to data. By assuming zero-mean additive white Gaussian noise on the parameters, the weighted sum of squared residuals, $\psi$, can be used as a placeholder for the likelihood. $\psi$ can be defined as

4 $$\begin{array}{*{20}c} {\psi \left( {{\varvec{\theta}}} \right) = \mathop \sum \limits_{i = 1}^{N_s } \left( {\frac{{C\left( {{{\varvec{\theta}}}, t_i } \right) - C_{M, i} }}{{\sigma_{M, i} }}} \right)^2 } \\ \end{array}$$

where $N_s$ is the number of datapoints, ${{\varvec{\theta}}}$ is the parameter vector, $\sigma_{M, i}$ is the standard deviation of the measurement error, $C\left( {{{\varvec{\theta}}}, t_i } \right)$ and $C_{M, i}$ are the simulated and measured concentrations at schedule time $t_i$, respectively. Then, likelihood-based confidence intervals can then be based on likelihood thresholds defined by the Chi-squared distribution $\chi$, with a confidence region

5 $$\begin{array}{*{20}c} {\left\{ {{{\varvec{\theta}}}{|}\psi \left( {{\varvec{\theta}}} \right) - \psi \left( {{\hat{\varvec{\theta }}}} \right) < \chi^2 \left( {\alpha , df} \right)} \right\}} \\ \end{array}$$

where the confidence interval is constructed for a nominal parameter set ${\hat{\varvec{\theta }}}$ (Raue et al. 2009). In this research, a quantile of $\alpha$ = 0.68 and $\# dof$ = 1 as in (Raue et al. 2009) were used to construct point-wise confidence intervals for each parameter $\theta_i$. The parameter $\theta_i$ is considered practically identifiable when the confidence interval is finite. Henceforth, when $CI_{crit}$ from Eq. 3 is calculated using the confidence interval from profile-likelihood, it will be referred to as $PLB_{crit}$ to show that it was calculated through the profile likelihood bounds. In contrast, when $CI_{crit}$ is calculated with comparable Monte Carlo quantile bounds ($Q_{\theta_i , 0.84}$ and $Q_{\theta_i , 0.16}$, the 84th and 16th quantiles to also capture 0.68 of the cluster), it will be referred to as $QB_{crit}$.

Both $PLB_{crit}$ and $QB_{crit}$ enable quantitative measurement of parameter uncertainty, which allows for direct comparison of different sampling schedules. Confidence intervals have been used to compare model performance and measurement schemes (Simpson et al. 2020), and $PLB_{crit}$ uses these intervals to form a scalar performance measure. The metrics are minimised when confidence intervals or quantile bounds are narrower, which indicates that residual error rapidly increases as one moves away from the optimal parameter solution. Conversely, higher values of the metrics indicate that residual error increases only when moving much further away from the optimal parameters, which is symptomatic of parameter trade-off and practical non-identifiability issues.

### Genetic Algorithm Method

Prior to applying the genetic algorithm, a practical identifiability analysis was performed with profile-likelihood to ensure that parameter bounds would be finite and physically feasible. Then a relatively simple GA implementation was implemented to find the optimum sample placement (${{\varvec{S}}}_{opt}$). One hundred organisms (${{\varvec{S}}}_k$) were iterated upon in each generation, and they competed to improve the $PLB_{crit}$ metric in each generation.

The procedure used for the genetic algorithm is as follows:

1. Randomly select $k = \left\{ {1, 2, \ldots 100} \right\}$ initial sampling schedules (organisms) (${{\varvec{S}}}_k$).
2. Determine the $PLB_{crit}$ value for each organism ($PLB_{crit,k}^P$) and parameter scenario ($P$).
3. Across the considered parameter scenarios, save the worst-case (maximum) value for each organism: $PLB_{crit,k}^{max} = {\mathop {\max }\limits_P} (PLB_{crit,k}^P )$.
4. Re-order organism values (${{\varvec{S}}}_k^*$) in ascending order of $PLB_{crit,k}^{max}$, leading to the minimum value among the organisms being ranked the best**.**
5. Carry forward the best organisms to the next generation through a weighted cloning process.
6. Mutate (add noise) to the sample placement of all but the highest ranked organism (${{\varvec{S}}}_{2..100,} = {{\varvec{S}}}_{2..100}^* + {\rm{\mathbb{N}}}\left( {0,16} \right)_{2..100}^{1..N_S }$).
7. Repeat steps 2–6 to simulate successive ‘generations’.

Some practical constraints were imposed on the sampling schedules throughout the genetic algorithm procedure. One data point was always placed at the $t_0$ = 0. A condition of $\Delta t > 5$ minutes was imposed on samples to represent the limitation of practical sampling. Additionally, the 5 min of time following the bolus input at t = 60 min were set as infeasible sampling times, due to a practical consideration of local mixing effects being present immediately post-bolus (Lam et al. 2021). In cases where the change would violate one of the constraints, the sampling point was shifted to the nearest valid location. MATLAB’s ‘lsqnonlin’ function was used to perform parameter identification through minimisation of $\psi ({{\varvec{\theta}}})$ given ${{\varvec{S}}}_k$. The lsqnonlin function with ‘StepTolerance’ = 1e−7, ‘OptimalityTolerance’ = 1e−7, and the other settings were left as default. Computational time was reduced for step 2 by running the 100 organisms through a parallel for-loop using MATLAB’s parallel computing toolbox.

Steps 3–4 implement a simple minimax scheme to consider the performance of multiple parameter scenarios in the optimal experimental design process. The true parameter values are not known in a practical setting, the worst-case behaviour under the range of representative scenarios is considered to address this issue. In contrast to a Bayesian modelling approach, this process does not require setting a prior distribution for the parameters, but it is limited to covering a finite number of feasible scenarios. Step 5 was performed through cloning organisms through to the next generation using the following function. Each j^th^ clone in the new generation was selected based on the previous generation’s ordered organisms:

6 $$\begin{array}{*{20}c} {{{\varvec{S}}}^{\varvec{*}} \left( j \right) = S\left( {{\text{ceil}}\left( \frac{j}{14} \right)^2 } \right)} \\ \end{array}$$

which cloned 14 of the best, 5 of the second best, 5 of the next, and so on until the last cloned organism in the new generation was the 52nd from the previous. For the mutation in step 6, except in the best sampling schedule from the generation ($j$ = 1), normally distributed noise ($\mu$ = 0, $\sigma$ = 16 [min]) was sequentially added to each sampling time following the initial t = 0 min sample. If the noise added to a particular sampling time contradicted the $\Delta t > 5$ min or post-bolus cooldown conditions, then the time was shifted to the nearest valid location.

The genetic algorithm was applied to each of the three cases for all three parameter scenarios. This was performed for a minimum number of samples $N_s$ = 3, 4, or 5 for cases 1–3 respectively, up to a maximum $N_s$ = 20. The algorithm was applied using a minimax design rule, in which the three parameter scenarios were optimised upon the same ${{\varvec{S}}}_K$ set simultaneously such that the maximum $PLB_{crit}$ value of the parameter scenario was minimised for each generation. 150 generations were run for each case to observe speed of convergence of the sampling schedules. All analyses were undertaken on an Intel core i7-9700 (@3.00Ghz) with 32 GB RAM and MATLAB (Version R2022a 64-bit).

The confidence interval of parameter values achievable with the proposed method was compared to the confidence intervals obtained by traditional time-uniform sampling methods. Such a direct comparison targets the metric of primary value in model-based analysis. Positive performance of the proposed algorithm will be evident in a consistent ability to lead to parameter values with a small confidence interval. Furthermore, to enable comparison of each of the scenarios (*P*), a second analysis was undertaken wherein the genetic algorithm was applied separately to each of the three scenarios. 100 generations were run for each individual scenario, and steps 3–4 of the genetic algorithm were simplified down to simply ordering the organisms by their minimum $PLB_{crit}$ values.

## Results

### Genetic Algorithm Results

**Fig. 2:**
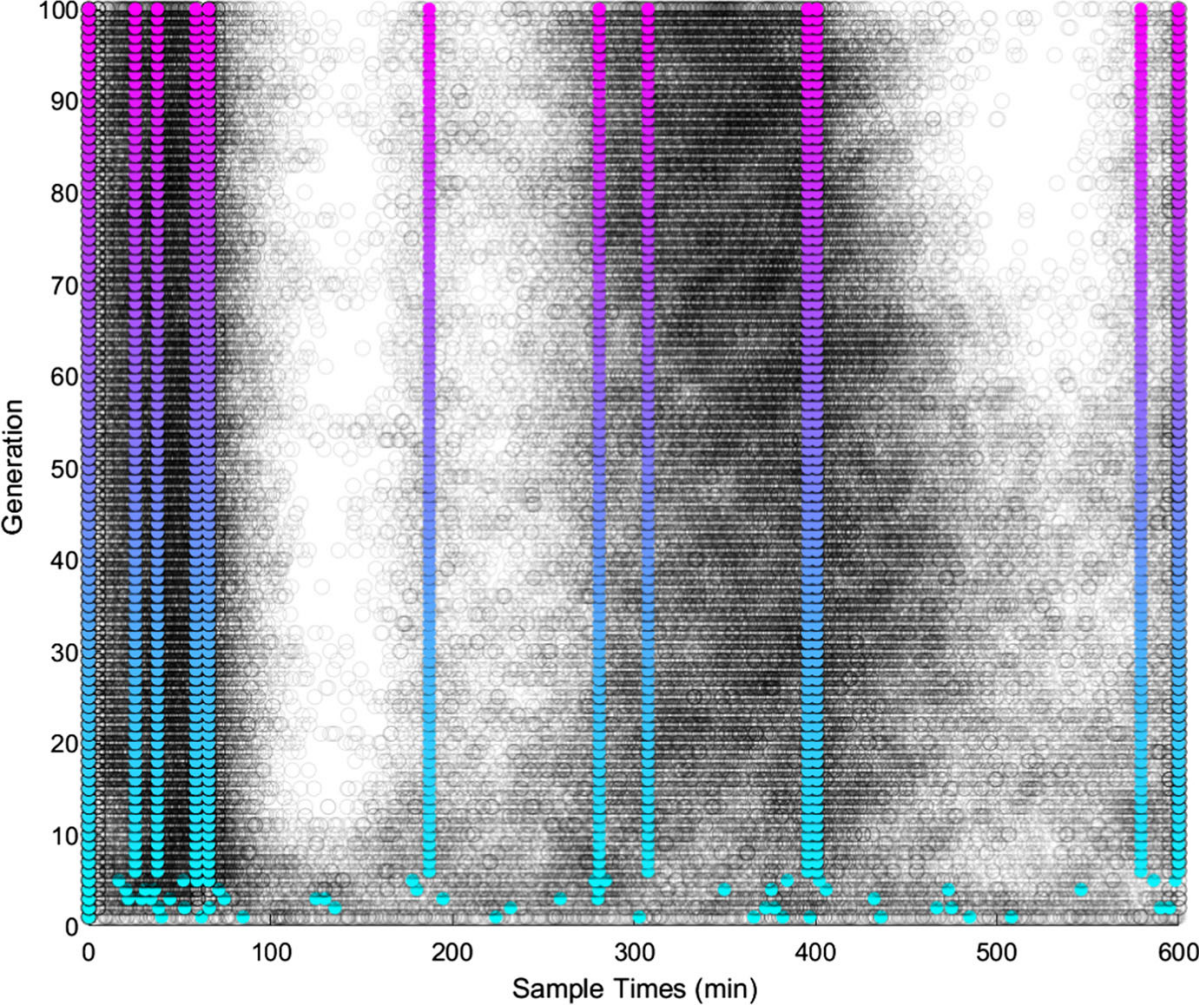
Genetic algorithm output over 100 generations of convergence for Model 1, parameter scenario 1, ${{\varvec{N}}}_{{\varvec{s}}}$ = 12. The organism with the lowest ${{\varvec{PLB}}}_{{{\varvec{crit}}}} ({{\varvec{S}}}_1^{\varvec{*}} )$ is plotted in colour, while the other 99 organisms are grey dots to show clustering (Color figure online)

**Fig. 3:**
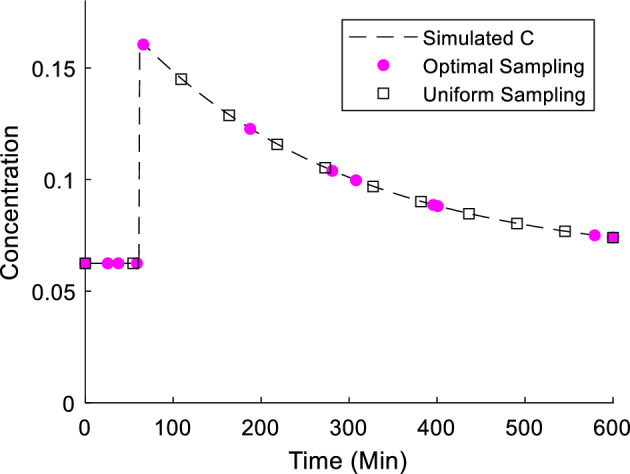
The optimal sampling points resulting from the genetic algorithm applied for Model 1, parameter scenario 1, ${{\varvec{N}}}_{{\varvec{s}}}$ = 12

**Fig. 4:**
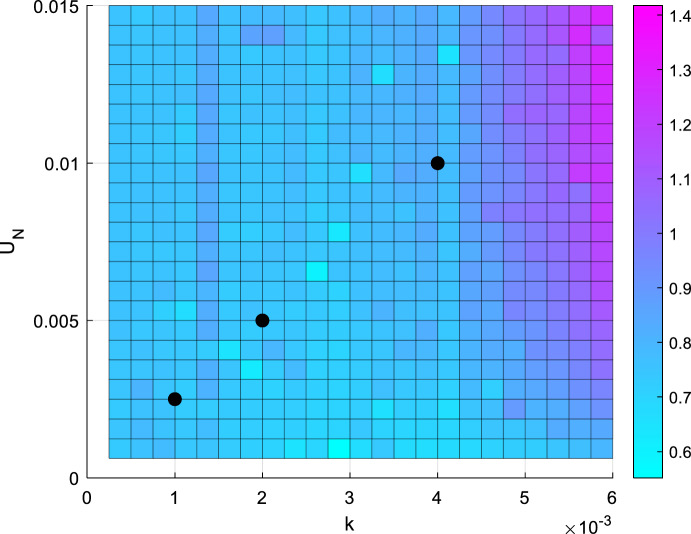
Validation of the genetic algorithm optimisation for ${{\varvec{N}}}_{{\varvec{s}}}$ = 10. The heatmap shows the ratio of optimised ${{\varvec{PLB}}}_{{{\varvec{crit}}}}$ to time-uniform ${{\varvec{PLB}}}_{{{\varvec{crit}}}}$, and the black dots from left to right represent parameter scenarios 1–3, respectively (Color Figure Online)

Across all of the tested sampling quantities, the genetic algorithm was able to locate sampling schedules with lower PL-measures than time-uniform sampling. Figure 2 shows the progression of 100 generations for $N_s = 12$ in case 2, parameter scenario 1, along with the sampling locations visualised on the simulated C-profile on Fig. 3. From generation six onwards, convergence can clearly be seen as the same sampling schedule continues to dominate successive generations. For Cases 1 and 2, results of the genetic algorithm are similar across all the cases and $N_s$. In data not shown, there was a general trend for slower convergence for larger $N_s$. To validate the usage of the minimax algorithm, the ratio of optimised $PLB_{crit}$ to $PLB_{crit}$ from sampling at equi-distant times was checked in a grid of parameter values. The optimised sampling outperformed the time-uniform sampling within the neighbourhood of the scenarios, and an example of one of the validation outputs for $N_s$ = 10 is shown in Fig. 4.

In the first generation of each Case I and II setting, the modified $QB_{crit}$ derived from Krausch et al. (2019) was calculated for comparison with $PLB_{crit}$. Values of the Q-criterion were strongly correlated with the PL-measure. Across all the simulated parameter scenarios and $N_s$, correlations in the range [0.893, 0.998], and [0.953, 0.996] were found for Cases 1 and 2 respectively. On average, the calculation of $PLB_{crit}$ was 25 to 36 times faster than $QB_{crit}$. Due to its stochastic nature, the values for $QB_{crit}$ fluctuated slightly between runs unless the MATLAB’s random number generator had a fixed at the start of simulations. On average, when considering a single parameter scenario, each generation of Case 1 and 2 took 0.38 and 0.77 s, respectively. Implementing the minimax algorithm increased computational time additively, i.e. considering the three scenarios increased the computational time threefold.

### Case 1 and 2 Results

**Fig. 5:**
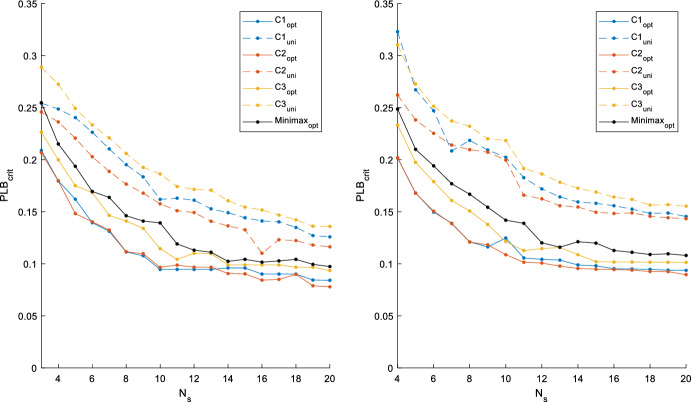
Tradeoff curves for the 2-parameter Case 1 (left) and 3-parameter Case 2 (right). The dotted lines represent the error when naive uniform sampling (uni) is implemented, while the solid lines represent the optimal sampling (opt) following optimisation of ${{\varvec{PLB}}}_{{{\varvec{crit}}}}$ via genetic algorithm (Color Figure Online)

The final results for the full range of $N_s$ tested for each case in Cases 1 and 2 are shown in Fig. 5. The methods were able to consistently reduce $PLB_{crit}$ for each of the three cases. Simulations for the full Case 1 and 2 trade-off curves took 15 and 21 min for each parameter scenario, respectively. Additionally, the minimax approach was able to locate sampling times that yielded sampling times with a lower $PLB_{crit}$ than the time-uniform sampling schedules of all three scenarios, except in the case of $N_s$ = 3 for Case 1. Compared to Case 1, values of $PLB_{crit}$ were consistently higher for Case 2, and the confidence intervals of individual parameters $k$ and $U_N$ (not pictured) were also greater for each parameter scenario.

### Case 3 Results

**Fig. 6:**
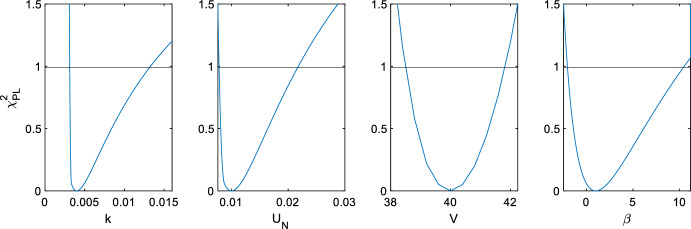
Profile likelihood analysis of Case 3, parameter scenario 1, for ${{\varvec{N}}}_{{\varvec{s}}}$ = 20. The threshold at ${{\varvec{\psi}}}$ = 0.99 indicates the bound for the pointwise confidence interval of each parameter. Note the scale of ${{\varvec{\beta}}}$ exceeds the physiologically feasible range

Within the local region of parameters and inputs that were tested, the lower confidence bound of $\beta$ fell below $\beta = 0$, indicating physically infeasible parameter values for Michaelis–Menten mechanics. Additionally, the upper bounds for the $k$ and $U_N$ parameters started tending towards infinity for small $N_s$ in parameter scenario 3. These practical identifiability issues were identified during the profile likelihood analysis performed prior to implementing the genetic algorithm, and the results of this analysis for Case 3, parameter scenario 1 are shown in Fig. 6. These identifiability issues persisted when modifications to the experimental design, such as doubling the bolus or halving the theoretical measurement noise, were attempted. Despite the identifiability issues, the genetic algorithm methods were attempted for parameter scenario 1, and they consistently yielded reductions in the $PLB_{crit}$ metric. However, the confidence intervals for the $\beta$ variable remained in infeasible regions, and the variance in $\beta$ dominated the $PLB_{crit}$ metric. Thus, the model of Case 3 failed the primary practical identifiability check that the proposed approach requires, hence the optimisation process is somewhat moot. The results of this analysis are presented in “Appendix”.

## Discussion

Through optimising $PLB_{crit}$, the GA was able to locate much better sampling schedules across the wide range of cases and parameter scenarios tested. For Cases 1 and 2, shifting from a uniform to an optimised sampling schedule led to an average reduction in $PLB_{crit}$ of 33.1% and 36.9%, respectively (see Fig. 5). Furthermore, Fig. 5 shows that the minimax optimisation of the three parameter scenarios yielded sampling protocols which still improved identifiability outcomes for all scenarios in all cases (with the single exception of $n_s$ = 3 in Case 1). The $PLB_{crit}$ metric achieved high correlations (mean correlations > 0.95) with a form of the $QB_{crit}$ metric previously established by Krausch et al. (2019), while also reducing computational time by an order of magnitude.

In addition to providing a clear path towards improving the placement of samples, the methodology allows for the clear visualisation of trade-off curves for guiding experimental design decisions. The relatively fast execution of the profile-likelihood values enables the comparison of optimal curves against the naive time-uniform sampling curve. The trade-off curves plotted in Fig. 5 allow for a clear comparison where one could either reduce the number of samples required while maintaining the same level of parameter certainty or improve the level of parameter certainty through optimising a fixed quantity of samples. The increase in parameter variability when moving from Case 1 to Case 2 is in agreement with the concept of parsimony in modelling—the increased complexity of the parameter identification led to the corresponding increase of $PLB_{crit}$.

Despite the increasing use of PL in practical identifiability analyses, there seems to have been little utilisation of PL for experimental design optimisation. Through sharing the statistical principles of the Monte Carlo methods, the $PL_{crit}$ is able to detect nonlinearities. However, rather than relying on the stochastic nature of the MC, the $PL_{crit}$ provides a deterministic metric that remains consistent between iterations. While relatively simplistic in its implementation, the GA used for this work was able to quickly locate sampling protocols that made clear improvements to identifiability outcomes. Practical identifiability concerns were also able to be addressed, as seen from the results of applying profile likelihood to Case 3. Additionally, several stages of the process were parallelisable: the calculation of each organism’s $PL_{crit}$ value was parallelised in this process, and further improvements could be made through calculating the scenarios of different $N_s$ values in parallel.

The methodology presented in this paper shares downsides common to other methods for optimal design of experiments. The optimisation of sampling was undertaken using a domain of suspected parameter values. Having knowledge of these local parameters would require either preliminary studies, or estimation via some indicative a priori information. This was partially addressed through using the minimax to address the differences between the three parameter scenarios without significant additional cost to computational time. However, this still assumed some level of a priori knowledge about how the local parameters were distributed. Additionally, the simulated nature of this methodology assumes that the model itself is accurate to measured phenomena. In the case that the model has mismatch or bias compared to the true data, the clustering of sampling around some perceived optimality could hinder the unique identification of parameters and mask the issue of model mismatch. However, if the model has previously undergone validation and the likely parameter scenarios are known, then the usage of this methodology would be justified (Wieland et al. 2021).

In cases where data could be limited in quantity due to cost of sampling, or a limited nature of the data (e.g., blood sampling), the methods could provide a means for maximising the information available within each sample. Furthermore, applying the methodology to generate the trade-off curves in Fig. 5 could provide guidance and evidence to support decisions regarding changes in sampling procedure. From the modeller’s perspective, the $PLB_{crit}$ metric allows for a reduction in computational requirements for experimental design, and the minimax approach allows for consideration of multiple parameter behaviours. Additionally, the level of complexity of the GA implemented in this paper has been kept relatively low to demonstrate the ease of implementation of the methods. While it is certainly possible to make further adjustments to the GA in order to improve the convergence speeds, it is not necessary to make such changes in order to achieve a near-optimal sampling protocol with current computational capabilities.

This research focussed on applying the $PLB_{crit}$ metric and GA approach to a PK-PD dose–response model. In the future, it would be valuable to test the method on a wider range of modelling contexts. This research has shown that it is not possible to locate an optimal sample timing common to all parameter scenarios. However, the minimax approach could lead to optimal sample timing schedules that lead to the best possible parameter confidence across the expected range of characteristics. Nonetheless, the likely parameter domain remains a critical input to this approach. Additionally, this work focussed on testing candidate sampling protocols on a continuous time measure. A more restricted region of sample timing (i.e. allowing discrete sampling locations on minutely grid points) could yield improvements in the GA convergence speed, while also aligning with practical limitations of physical data collection.

## Conclusion

This analysis considers the usage of a novel, PL based experimental design metric for optimising the identifiability of parameters with relatively low computational effort. A genetic algorithm was applied with this metric to optimise sampling protocols across a range of model complexities, parameter scenarios, and number of samples. The methods were demonstrated consistent reductions in parameter variance (~ 33%) across the parameters and scenarios explored, including an example in which three parameter scenarios had to be simultaneously optimised using minimax rule. The results showed clear trade-off curves that quantified the extent to which either parameter variance could be reduced, or numbers of samples could be reduced.

Overall, this analysis showed that it is possible to account for the nonlinear nature of models in MBDoE while maintaining reasonable computation times. The $PLB_{crit}$ metric provides adds a new alternative to the existing $QB_{crit}$ metric. By giving up a small amount of information regarding higher dimensional parameter constellations, the $PLB_{crit}$ metric reduces computational time by an order of magnitude, leaving more time available for running computationally intensive applications such as model optimisation via GA. However, it must be acknowledged the optimisation of sampling schedules through these methods, while mathematically useful, must be considered against the practical considerations of those implementing the experiments.

## Figures and Tables

**Fig. 7:**
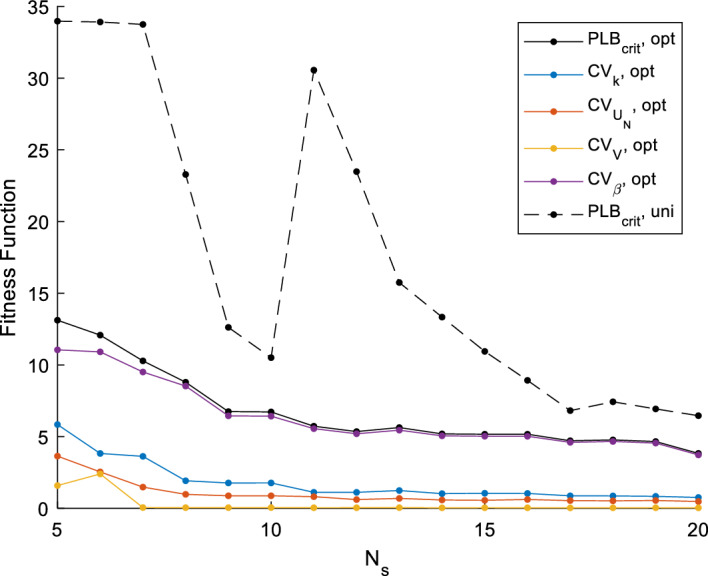
Trade-off curves for the ${{\varvec{PLB}}}_{{{\varvec{crit}}}}$ values of optimised and time-uniform sampling locations for Case 3, parameter scenario 1, alongside optimised coefficient of variation (CV) values for individual parameters (Color Figure Online)

## Appendix

Figure 7 shows the results of optimising sampling schedules for Case 3. In all cases, the lower bound of the $\beta$ parameter was negative, indicating physically infeasible values for Michaelis Menten mechanics. Additionally, the $PLB_{crit}$ metric was mainly influenced by the variance in the $\beta$ bounds. Coefficient of variation values for parameters were calculated using the profile likelihood bounds with the following definition:

$$CV_{\theta_i } = \frac{\theta_i^+ - \theta_i^- }{{2\theta_i^* }}.$$
